# Pyloric and antral strictures following corrosive acid ingestion: A report of four cases

**DOI:** 10.4103/0971-9261.71749

**Published:** 2010

**Authors:** Ram Mohan Shukla, Madhumita Mukhopadhyay, B. B. Tripathy, K. C. Mandal, B. Mukhopadhyay

**Affiliations:** Department of Pediatric Surgery, N. R. S. Medical College and Hospital, Kolkata 14, India; 1 Department of Pathology, IPGMER, Kolkata 20, India

**Keywords:** Acid ingestion, acquired antral stricture, acquired pyloric stricture, gastric outlet obstruction, gastrojejunostomy

## Abstract

This study reports four children who developed complete stricture of pylorus and antrum of the stomach following accidental ingestion of corrosive agent (toilet cleaner).

## INTRODUCTION

Ingestion of corrosive agents is not an uncommon cause of benign strictures of the upper aerodigestive tract in India. Easy availability of hydrochloric acid in the form of a cheap toilet cleaner is a frequent cause of pyloric and antral strictures. There are only few reports published highlighting the management of corrosive stricture of pylorus and antrum of the stomach in children.[1] In a study of 220 cases, 52 patients ingested acid agents and 2 of them (3.8%) presented with gastric outlet obstruction without esophageal stricture.[2]

## CASE REPORT

Four children (three boys and one girl), all with a common history of ingestion of toilet cleaner (hydrochloric acid), were admitted in our department and managed successfully. Ages of the patients were 1 year 7 months, 4 years, 5 year 4 months, and 9 years. All of them took it accidentally. Exact amount of acid ingested was not available. The patients were treated conservatively in peripheral hospitals (intravenous fluid, antibiotics, H2 receptor blockers, and steroids) before being referred to us for surgical intervention. Mean duration of conservative treatment was 4 weeks (range from 3 to 6 weeks). Three patients presented with nonbilious vomiting after each feed and only one patient was tolerating small amount of liquid.

The mean body weight before acid ingestion was 15 kg (range, 8–30 kg) and average weight at the time of surgical intervention was 9.25 kg (range, 5–18 kg) with a mean weight loss was 38% (range, 33–40%). All these patients were nutritionally depleted. Clinical examination of abdomen revealed epigastric fullness in two patients. Near complete gastric outlet obstruction was seen in all cases in upper gastrointestinal contrast study. Gastroduodenoscopy revealed stricture of pylorus in three patients and antral stricture in one case. Oesophagus was normal in all the cases. Endoscopic biopsy showed evidence of fibrosis and inflammation. After correction of dehydration, the patients were operated.

Laparotomy revealed complete obstruction of pylorus in three patients and antrum in one patient. One patient had antral stricture where we could pass only 10 size Ryle’s tube. Posterior gastrojejunostomy was performed in the relatively healthier upper part of the stomach. Postoperative period was uneventful in all four cases. There was rapid recovery and steady gain in body weight in all four patients. Mean duration of follow-up was 20.5 months (range, 4–42 months). Follow-up upper gastrointestinal endoscopy was normal in all the cases.

## DISCUSSION

Easy availability of cheap toilet cleaner makes hydrochloric acid the most frequent cause of corrosive injury in India[3] The tendency of acids “ to lick the esophagus and bite the pyloric antrum ” is well known. Viscosity and specific gravity of corrosive acids are lower than that of liquid alkalis, hence acids are associated with rapid transit through the esophagus and the damage primarily occurs in the antrum and pyloric region of the stomach.[4] Antral spasm also causes pooling of the corrosive and more damage to the antrum. Another reason for greater susceptibility of stomach is its columnar epithelium whereas esophagus has a more resilient squamous epithelium.[5] The degree of mucosal injury depends on the nature of the agent, the amount and concentration ingested, the amount of food in the stomach at the time of ingestion and the mode of ingestion.

Vomiting, rapid loss of body weight, and decreased oral intake remain the most notable features after acid burns in children.[6]

Timing of surgery is controversial, but early surgical intervention remains the treatment of choice.[2,7] Feeding jejunostomy and endoscopic balloon dilatation of stricture,[8] gastrojejunostomy with or without vagotomy,[9] pyloroplasty,[6] or antrectomy with Bilroth I anastomosis[10] are the various options available to us. Each procedure has got its merits and demerits. Partial obstruction with moderate mucosal injury usually responds to pyloroplasty.[6] Gastric resection is a major surgery in nutritionally depleted patients and has its associated morbidities. Poor nutritional status and extensive perigastric adhesions are indications of gastrojejunostomy in our patients. Diminution of acid and pepsin production due to damage of glandular elements would weigh against the addition of vagotomy in addition to the drainage procedure.[11] Early surgical intervention resulted in a satisfactory recovery in all four cases with a mean weight gain of 95% (range,50% to 220%) at the last follow-up. There were no symptoms of vomiting or postprandial fullness. Follow-up upper gastrointestinal endoscopy did not reveal any abnormality after 1 year of surgery.

There is a great need for adult education and for legislation to ensure correct labeling, safe packaging in child proof containers and to restrict the strength of caustic agents. Early surgical intervention, individualized according to the site and extent of damage gives excellent result. Gastrojejunostomy is a very safe operation with minimum morbidity and excellent long-term outcome.
